# Estimation and linkage between behavioral problems and social emotional competence among Pakistani young school children

**DOI:** 10.1371/journal.pone.0278719

**Published:** 2023-05-25

**Authors:** Arooj Najmussaqib, Asia Mushtaq

**Affiliations:** Department of Applied Psychology, National University of Modern Languages, Islamabad, Pakistan; Chiang Mai University, THAILAND

## Abstract

Behavioral problems are commonly occurring concerns in school children and if left unidentified can result in worse outcomes in any society. The research aims to explore the prevalence of behavioral problems and its association with social emotional competence in young school children from a community sample of Islamabad, Pakistan. The cross-sectional study was conducted from April to June 2021 in four public primary schools in Islamabad, Pakistan. Two stage cluster sampling was used to select study sites. The sample comprised 426 school children (males = 182, females = 195) aged 4–8 years (Mean age = 6.5, SD = 1.09), from three different grades kindergarten, 1, and 2, respectively. The Child Behavior Checklist (CBCL) and Social Emotional Development Assessment (SEDA) were used to screen behavioral problems and social emotional competences of children. Data were analyzed using Stata 17. Prevalence for overall behavioral problems accounted for 65.4% (4–6 years) and 36.2% (6–8 years) in the abnormal (borderline and clinical) ranges of total problems. Social emotional competence scores were found significantly negatively associated with behavioral problems of children. The high prevalence necessitates the provision of mental health care to school-aged children. The findings should be taken as a call to Pakistan’s policymakers, clinicians, and researchers to develop proper screening and management protocols for early intervention.

## Introduction

Mental illnesses in children have been identified as a significant public health concern and one of the leading causes of disability and economic costs to society [1]. Suffering, functional impairment, stigma and discrimination, and an increased risk of premature death are all associated with mental health problems [2,3]. According to global epidemiological data, 13 to 23% of children and adolescents have a mental disorder [4,5]. Early age mental health problems (MHP) endanger a child’s life. Therefore, during the last few decades, researchers’ focus has shifted from adults to preschool and young children’s mental health prevalence [6].

Numerous studies on young children indicate that mental health problems show remarkable stability and strongly predict mental disorders in adulthood [7]. In addition, Basten et al. (2016) [8] showed that a heterotypic continuity (i.e., one disorder predicting another at a later time point) of symptom patterns is common as children age. In a recent national survey of United States [9], mental health disorders were reported in 41% of children aged six to eleven. According to the findings, preschool children had a comparatively high rate of borderline behavior problems (46.5%). One in eighteen (5.5%) preschool children were identified with a mental disorder, with higher rates in males (6.8%) than in females (4.2%). Researchers have categorized behavioral problems as internalizing and externalizing problems. Internalizing problems are defined as anxious and depressive symptoms, social withdrawal, and somatic complaints. Externalizing problems on the other hand are categorized as aggressive, oppositional, and delinquent behavior. Children having internalizing or externalizing symptoms may have challenges as long-term consequences within social, academics and later professional environment [10]. Alarmingly, internalizing problems have steadily increased in children, up from 4.3% in 1999 and 3.9% in 2004 to 5.8% by 2017 [11]. Furthermore, children with externalizing problems significantly predict Internet Gaming disorder, whereas internalizing problems predict Internet Addiction [12,13].

In Pakistan, mental health services are the most neglected, with 10–16 percent of the population, or more than 14 million people, suffering from mild to moderate mental diseases [14,15]. Data from limited studies conducted in Pakistan using different assessment measures revealed that approximately 34.4% of school children suffer from mental health problems [16,17]. A recent telephonic survey [18] from parents using strengths and difficulty questionnaire (SDQ) showed a 15.9% prevalence of overall behavior problems in children aged 6–16 years. Furthermore, estimates for conduct problems were about 26.6%, emotional problems 22.5%, peer problems 13%, hyperactivity 10.6%, and social problems 3%. Another research with preschool children [19] observed a significant percentage of borderline behavior problems (46.5%). Author reported significant gender differences, with males scoring higher on externalizing problems than females. However, there is no empirical evidence of using teachers’ reports in a cross-sectional study design to investigate emotional and behavioral difficulties in young school children aged 4–8 years.

In addition to the concerns about behavioral problems, these issues may manifest as a result of children’s own individual difficulties with social emotional competence [20]. Social emotional skills are strongly associated with children’s trajectory of development of internalizing and externalizing problems during early childhood [21]. Considerable literature demonstrates that children’s social emotional competence serves as potential protective factors for challenging life events and are not limited to immediate wellbeing [22]. Recent studies in several Asian countries recommended and highlighted the importance of school based mental health care to develop social emotional skills in children [23]. Teaching social emotional skills such as emotions understanding and regulation, prosocial behaviors, and social skills at early age have found to be improving learning behavior, wellbeing, inattention, aggression and depressive/withdrawn behaviors [24,25]. Experts from Pakistan also recommended need of social emotional skills development of children as a remedy to reduce behavioral problems [18,19]. However, not much research work is done in the respective area of social emotional competence. Due to the scarcity of local research with respect to young school children, our primary goal in this study was to assess the estimates of internalizing and externalizing problems in young school children. Another aim of this study was to assess the associations between social emotional competence, internalizing and externalizing problems (IEPs) in young Pakistani school children aged 4–8 years.

## Method

The present study was part of a PhD research project. Inclusion criteria was based on curriculum age group(4–8 years) comprised of males and females.

### Study design

The present study is based on cross sectional study design. It was carried out in April–June 2021 in four public sector schools of Islamabad, Pakistan after obtaining ethical approval from board of studies of National Language of Modern Languages, Islamabad and official permission from Federal Directorate of Education, Islamabad.

### Participants

The target population was school children aged 4–8 years as per the requirement of the intervention curriculum. Therefore, inclusion criteria were age range of 4-8years, both males and females enrolled in public schools from three grade levels, i.e., kindergarten, grade one, and grade two. A sample of 426 eligible students (48.48% males) was drawn from 15 classes and four public schools.

### Sampling and procedure

Two staged cluster sampling technique was used to induct schools and study participants. As a first step, permissions were sought from the Federal Directorate of Education (FDE), Islamabad. The permission application took 6.5 months due to covid-19 as initial lockdowns were occurring all over Pakistan, including Islamabad. Since the present study was part of intervention project, only primary schools’ data was requested from Federal Department of Education. The permission was granted in November 2020 to conduct the study, and FDE nominated four public primary schools in Islamabad. As a second step, meetings were arranged with school principals to discuss the administrative requirements. They nominated fifteen teachers from respective classes for the training and facilitation during the research project. Written consent was taken from the teachers, and due to covid restrictions of social and public meetings, parents were approached through letters from the schools. Trained research assistants administered a child report instrument in small groups. Another set of assessment protocol was completed by teachers for each child using pen and paper method.

A statistical power analysis was performed using G-power software for sample size estimation, based on data from the meta-analysis [26], comparing school-based studies for depression and anxiety programs. With an estimated small effect size [27] of 0.25, an alpha = .05, and power = 0.80, the projected sample size was 98. Hence, the initial total sample size of N = 464 was deemed adequate for the main objective of this study and should also allow for expected attrition. The current study’s sample consisted of 426 school children (males = 206, females = 220) recruited from four public sector schools in Islamabad, Pakistan. Children were from 4-8years of age (Mean age = 6.3 years, SD = 0.84) belonging to three classes Kindergarten, grade one, and grade two, respectively. 24 teachers (two from each class as per nominated by school administration) completed the outcome measures.

### Measures

Behavioral problems were measured using teacher reported Urdu versions of Child Behavior Checklist (CBCL) [28]. Whereas, social emotional competence was assessed using translated version of child reported measure of Social Emotional Development Assessment (SEDA) [29].

1. **Demographic form.** A form was developed to obtain data about the sample’s various demographic variables including gender, age, family income and family system.
2. **Child behavior checklist (11/2-5)-CTRF.** Children (aged 4–5 years) behavior problems were measured through the Teacher Reported Urdu Version of Child Behavior Checklist (CBCL-CTRF) [29], 99 items. It has six empirically based syndrome scales: Emotionally Reactive, Anxious/Depressed, Somatic Complaints, Withdrawn, Attention Problems, and Aggressive Behavior. These syndrome scales broadly form two subcategories of behavioral problems, namely “internalizing” and “externalizing.” Scoring is done on 3-point scale, where 0 = not true, 1 = sometimes true, and 2 = often true or very true. Raw scores were converted to T scores and percentiles as per the scoring criteria. The Cronbach’s alpha coefficient of the original measure was .88 for the total problem scale and .89 and .77 for externalizing and internalizing subscales, respectively.
3. **Child behavior checklist (16–18)-TRF.** This teacher reported Urdu version of CBCL- TRF was used for children above five years of age. It is also a Likert-type scale comprised of 112 items scoring on 3-point scale, where 0 = not true, 1 = sometimes true, and 2 = often true or very true. There are eight empirical-based syndrome scales named Anxious/Depressed, Withdrawn/Depressed, Somatic Complaints, Social Problems, Thought Problems, Attention Problems, Rule Breaking Behavior, and Aggressive Behavior [28]. This scale also has two broader categories of internalizing and externalizing problems. Raw scores were converted to T scores and percentiles as per the scoring criteria. The original measure’s Cronbach’s alpha coefficient was.97 for the total problem scale and.95 and.90 for the externalizing and internalizing subscales, respectively. Both scales’ validity is extensively established around the world.
4. **Social Emotional Development Assessment (SEDA).** Social Emotional Development Assessment (SEDA) [29] scale consisting of 12 self-report items rated on a 0–2 (i.e., “not true or rarely true” as indicated by a thumbs down clip art, “sometimes true” as indicated by a sideways thumbs clip art, “usually or always true” as indicated by a thumbs up clip art) that are used to assess social emotional skills in children from kindergarten to 2^nd^ grade across five domains: self-regulation, social skills, school belongingness, social responsibility, and optimism. Where, school belongingness and optimism have 3 items each and rest of the domains have 2 items (e.g., I wait my turn in line, I invite kids to play with me, I like myself). The scale evidenced adequate internal consistency (α = 0.83).

### Analytical plan

All analyses were conducted using Stata 17.0 [30]. Descriptive statistics were computed including frequencies, percentages for all problems for different categories (e.g., borderline and clinical). These results provided details for the estimates of the internalizing and externalizing problems in children. We than examined bivariate correlations to assess the associations between study variables such as behavioral problems (IEPs) and social emotional competence (SEDA).

The initial analysis for each dependent variable examined the main effects for the four independent variables of age groups (1 = 4+, 2 = 5+, 3 = 6+ and 4 = 7+ years) using univariate analysis of variance (ANOVA). Partial eta squared was selected for measuring the effect size. Then, multiple linear regression analyses were done to assess the associations between the SEC and IEPs.

### Ethical considerations

Initially, permission was sought from the Federal directorate of Education, Islamabad, to conduct the study. Nominations for schools were received, and teachers were later asked to provide the consent form and demographic information form to the parents. They were informed about the goal of the research and ensured that the information would only be utilized for research reasons. Class teachers who had been supervising the children for at least six months were asked to rate the children’s behaviors in class and during school hours using the respective scales followed by consent. All the information is coded and all the identifiable information has been removed from the dataset to protect the participants’ individual privacy.

## Results

The present study aimed at exploring prevalence and estimation of behavioral problems and its association with social emotional competence in young children of Pakistan. For this purpose, 426 [males:206 (48.4%), females:220 (51.6%)] participated from three different grades, i.e., Kindergarten (53.3%), 1st (30.8%) and 2nd (16%) respectively. The average age of the sample group is 6.3 (SD = 0.84) years. The mean individual household income was 25,365 PKR (SD = 11,527) (approximately USD-100) having nuclear family structure (62.4%). Table 1 presents the psychometric properties of the measures used in the study, whereas Table 2 presents the estimates of internalizing, externalizing and total problems of children of both age groups and gender.

**Table 1:** Psychometrics of CBCL-CTRF (n = 78), CBCL-TRF (n = 348) and SEDA (N = 426).

					Range			
Variables	No of items	M	SD	α	Potential	Actual	Skew	Kurt
**CBCL(11/2-5)- CTRF**								
Internalizing problems*	32	18.27	10.743	0.834	0–64	0–48	-0.009	-0.561
**Emotionally Reactive**	7	4.01	2.789	0.732	0–14	0–11	0.300	-0.646
**Anxious/Depressed**	8	4.90	3.234	0.737	0–16	0–14	0.348	-0.359
**Somatic complaints**	7	3.73	2.719	0.720	0–14	0–10	0.288	-0.972
**Withdrawn**	10	5.63	3.561	0.720	0–20	0–15	0.222	-0.385
Externalizing problems*	34	18.10	11.295	0.883	0–68	0–52	0.369	-0.175
**Attention problems**	9	5.32	3.547	0.736	0–18	0–15	0.442	-0.085
**Aggressive behavior**	25	12.78	8.360	0.734	0–50	0–37	0.287	-0.420
**Other problems**	34	18.18	11.582	0.741	0–68	0–48	0.238	-0.692
Total*	100	54.55	32.200	0.879	0–200	0–148	0.128	-0.446
**CBCL(6–18)- TRF**								
Internalizing problems*	27	8.94	8.935	0.827	0–54	0–38	1.077	0.350
**Anxious/Depressed**	16	5.48	5.619	0.748	0–32	0–24	1.099	0.438
**Withdrawn/depressed**	8	2.63	2.820	0.746	0–16	0–15	1.348	1.937
**Somatic complaints**	3	0.84	1.358	0.822	0–6	0–6	1.912	3.514
Externalizing problems*	32	9.81	11.830	0.911	0–64	0–52	1.380	0.906
**Rule-breaking**	12	3.40	4.391	0.759	0–24	0–19	1.438	1.178
**Aggressive behavior**	20	6.41	7.793	0.756	0–40	0–33	1.390	0.947
**Other problems**	6	2.18	2.436	0.747	0–12	0–10	1.270	0.878
**Social problems**	11	3.85	4.329	0.759	0–22	0–19	1.213	0.705
**Thought problems**	10	2.73	4.025	0.769	0–20	0–16	1.469	1.143
**Attention problems**	26	9.74	9.129	0.908	0–52	0–41	1.095	0.639
Total*	112	37.26	38.084	0.796	0–224	0–163	1.249	0.657
**SEDA**								
**Self-Regulation**	2	3.66	0.691	0.850	0–4	0–4	-2.324	5.982
**Social skills**	2	3.68	0.705	0.842	0–4	0–4	-2.523	6.879
**School belongingness**	3	5.33	0.908	0.743	0–6	0–6	-1.295	1.347
**Social responsibility**	2	3.65	0.820	0.850	0–4	0–4	-2.587	6.599
**Optimism**	3	5.75	0.603	0.678	0–6	0–6	-2.537	6.532
**Total**	12	22.07	2.451	0.713	0–24	0–9	-1.803	4.199

M = mean; SD = standard deviation; α = reliability coefficient; Skew = skewness; Kurt = Kurtosis.

* Scores are based on T-scores.

**Table 2:** Estimates of internalizing and externalizing problems in young school children (N = 426).

Variables	Normal			Borderline			Clinical		
**CBCL(1 1/2-5)- CTRF**	**Males** **n (%)**	**Females** **n (%)**	**Total** **n (%)**	**Males** **n (%)**	**Females (%)**	**Total n (%)**	**Males** **n (%)**	**Females** **n (%)**	**Total** **n (%)**
**Internalizing Problems**	12(15.4)	13(16.7)	25(32.1)	4(5.1)	3(3.8)	7(9)	21(26.9)	25(32.1)	46(59)
**Emotionally Reactive**	22(28.2)	22(28.2)	44(56.4)	7(9)	11(14.1)	18(23.1)	8(10.3)	8(10.3)	16(20.5)
**Anxious/Depressed**	24(30.8)	26(33.3)	50 (64.1)	5(6.4)	9(11.5)	14(17.9)	8(10.3)	6(7.7)	14(17.9)
**Somatic complaints**	17(21.8)	15(19.2)	32(41)	3(3.8)	2(2.6)	5(6.4)	17(21.8)	24(30.8)	41(52.6)
**Withdrawn**	30(38.5)	24(30.8)	54(69.2)	3(3.8)	15(19.2)	18(23.1)	4(5.1)	2(2.6)	6(7.7)
**Externalizing Problems**	24(30.8)	15(19.2)	39(50)	4(5.1)	12(15.4)	16(20.5)	9(11.5)	14(17.9)	23(29.5)
**Attention problems**	35(44.9)	34(43.6)	69(88.5)	1(1.3)	4(5.1)	5(6.4)	1(1.3)	3(3.8)	4(5.1)
**Aggressive behavior**	28(35.9)	32(41)	60(76.9)	8(10.3)	9(11.5)	17(21.8)	1(1.3)	0(0)	1(1.3)
**Total**	17(21.8)	10(12.8)	27(34.6)	2(2.6)	3(3.8)	5(6.4)	18(23.1)	28(35.9)	46(59)
**CBCL(6–18)- TRF**									
**Internalizing Problems**	96(27.6)	113(32.5)	209(60.1)	9(2.6)	21(6)	30(8.6)	64(18.4)	45(12.9)	109(31.3)
**Anxious/Depressed**	115(33)	138(39.7)	253(72.7)	18(5.2)	21(6)	39(11.2)	36(10.3)	20(5.7)	56(16.1)
**Withdrawn/depressed**	139(39.9)	150(43.1)	289(83)	22(6.3)	20(5.7)	42(12.1)	8(2.3)	9(2.6)	17(4.9)
**Somatic complaints**	144(41.4)	162(46.6)	306(87.9)	22(6.3)	13(3.7)	35(10.1)	3(0.9)	4(1.1)	7(2)
**Externalizing Problems**	113(32.5)	104(29.9)	217(62.4)	15(4.3)	17(4.9)	32(9.2)	41(11.8)	58(16.7)	99(28.4)
**Rule-breaking**	131(37.6)	121(34.8)	252(72.4)	11(3.2)	23(6.6)	34(9.8)	27(7.8)	35(10.1)	62(17.8)
**Aggressive behavior**	133(38.2)	140(40.2)	273(78.4)	20(5.7)	17(4.9)	37(10.6)	16(4.6)	22(6.3)	38(10.9)
**Social problems**	115(33)	126(36.2)	241(69.3)	17(4.9)	21(6)	38(10.9)	37(10.6)	32(9.2)	69(19.8)
**Thought problems**	115(33)	123(35.3)	238(68.4)	16(4.6)	16(4.6)	32(9.2)	38(10.9)	40(11.5)	78(22.4)
**Attention problems**	164(47.1)	160(46)	324(93.1)	3(0.9)	11(3.2)	14(4)	2(0.6)	8(2.3)	10(2.9)
**Total Problems**	112(32.2)	110(31.6)	222(63.8)	20(5.7)	22(6.3)	42(12.1)	37(10.6)	47(13.5)	84(24.1)

### Psychometrics of the scales

The psychometrics of the scales used in the present study are presented in Table 1. The mean score on a total of the CBCL- CTRF was 54.55 (SD = 32.2). The mean score on internalizing and externalizing scales was 18.27 (SD = 10.74) and 18.1 (SD = 11.29) respectively. Whereas the mean score on the total of the CBCL- TRF was 37.26 (SD = 38.08). The average score on internalizing and externalizing scales was 8.94 (SD = 9.93) and 9.81 (SD = 11.83) respectively.

Table 1 indicates alpha co-efficient and skewness of Urdu version of CBCL-CTRF, CBCL-TRF and SEDA. Internal consistency was high for both total scales and all subscales, implying that the scales are relevant and acceptable for Pakistani school children.

### Prevalence of behavioral problems

The present study sample showed that 65.4% children (4–6 years) and 36.2% children (6–8 years) have total behavioral problems as assessed by CBCL. For internalizing problems, estimates were 68% for younger children (4–6 years) and 39.9% for older children (6–8 years). For externalizing problems, estimates were 50% for younger children (4–6 years) and 37.6% for older children (6–8 years) Table 2 shows the categorization of both age groups and gender.

Further categorization of children was done based on the teacher’s rating on CBCL scales. Among children aged 4–6 years, 6.4% were categorized as having borderline behavioral problems, while 59% were rated in the clinical range in total problems. On the internalizing subscale, 9% of children were borderline, 59% were placed in the clinical range. On externalizing subscale, 20.5% of children were categorized as borderline, and 29.5% were reported in the clinical range.

Additionally, males showed higher abnormal (borderline and clinical) ratings in the overall internalizing domain (32%) with higher anxious/depressed domain, whereas; females were found higher in the somatic and withdrawn behaviors. Both showed equal in emotional reactivity. On the other hand, females were reported as having higher abnormal (borderline and clinical) in both overall externalizing (33.3%) and total problem scores (39.7%).

**Fig 1:**
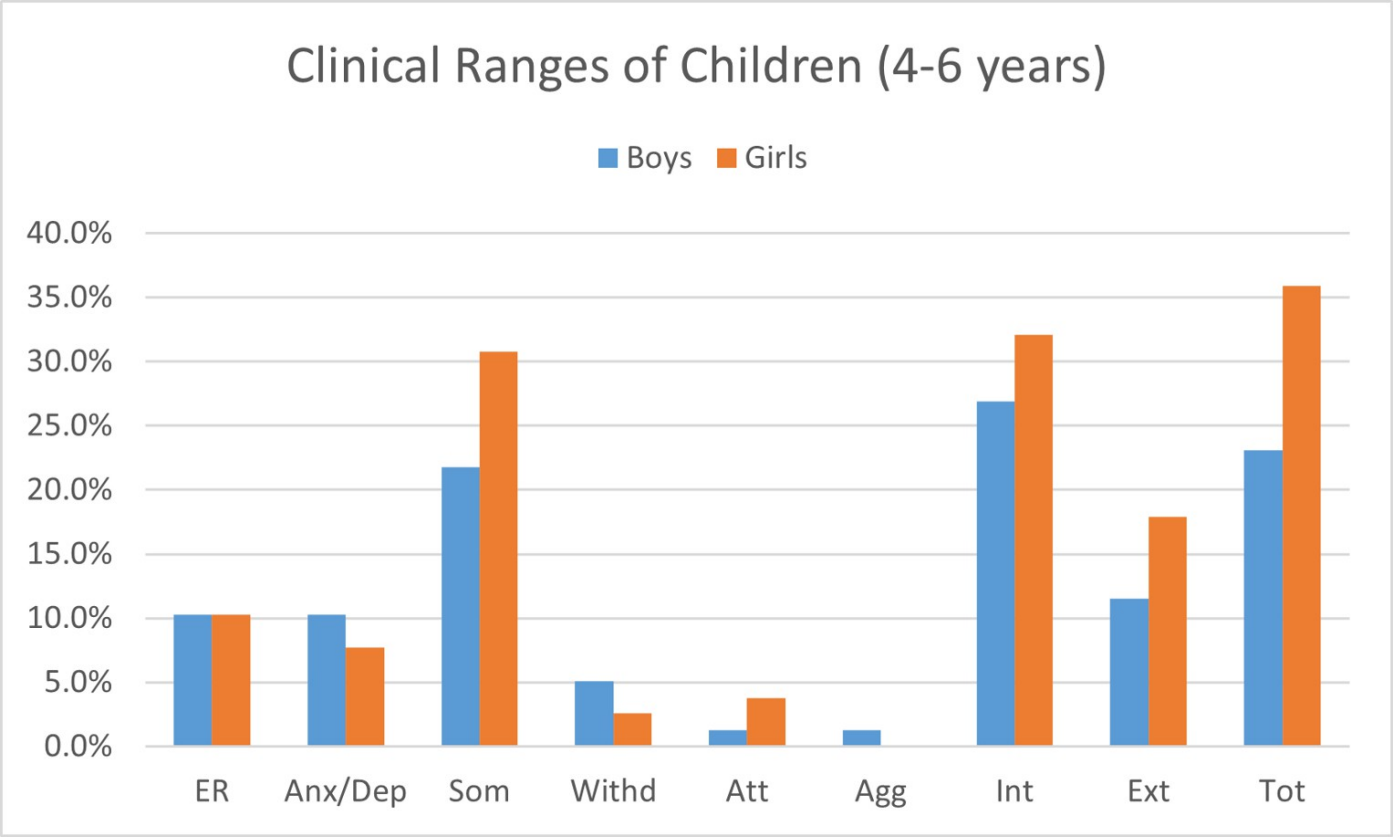
Clinical Ranges of males and females aged 4–6 years on CBCL- CTRF. ER = Emotionally reactive, Anx/Dep = Anxious/Depressed, Som = Somatic complaints, Withd = Withdrawn, Att = Attention problems, Agg = Aggressive, Int = Internal problems, Ext = External problems, Tot = Total problems.

**Fig 2:**
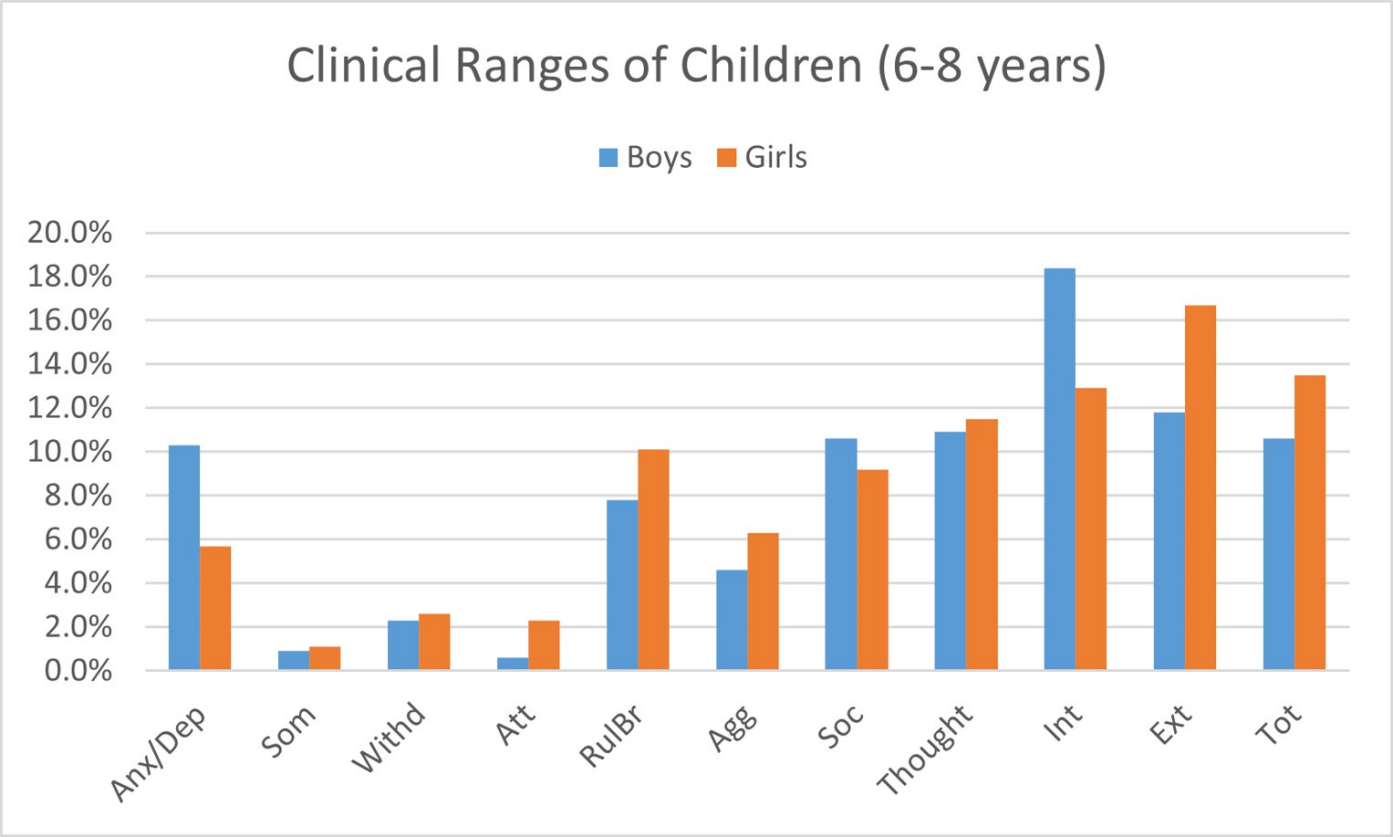
Clinical Ranges of males and females aged 6–8 years on CBCL- TRF. Anx/Dep = Anxious/Depressed, Som = Somatic complaints, Withd = Withdrawn, Soc = Social problems, Though = Thought problems, Att = Attention problems, Rulbr = Rule breaking, Agg = Aggressive, Int = Internal problems, Ext = External problems, Tot = Total problems.

Among children aged 6–8 years, 12.1% were categorized as having borderline behavioral problems, while 24.1% were rated in the clinical range in total problems. On the internalizing subscale, 8.6% of children were borderline, and 31.3% were ranked in the clinical range (see Table 2). On externalizing subscale, 9.2% of children were categorized as borderline, and 28.4% were reported in the clinical range. According to teacher reports, Figs 1 and 2 provides an illustrative view of percentages for clinical ranges of males and females.

In the higher age group (6–8 years), males again showed higher abnormal (borderline and clinical) ratings in the overall internalizing domain (21%) with higher anxious/depressed, somatic and withdrawn behaviors. Furthermore, males showed slightly higher abnormal (borderline and clinical) ratings in the overall internalizing domain (21%) with more elevated anxious/depressed, withdrawn and somatic complaints subscales.

On the other hand, females were reported as having higher abnormal (borderline and clinical) in both overall externalizing (21.6%) and total problem scores (19.8%). Females were also reported to have higher abnormal ratings(borderline and clinical) in rule breaking (16.7%), aggressive (11.2%), thought (16.1%), and attention problems (5.5%) than males.

The Table 3 and show the mean differences in the scores of study variables based on child gender, and age. Only significant mean difference is found in internalizing problems, where males have significantly higher internalizing problems than females. Table 4 represents the mean differences between the age groups with Post hoc analyses (LSD). Age group 1 (4 years + age) have more internalizing problems than older children. For externalizing problems, children with age range 5–5.11 years have significantly high mean scores, 5+ aged children have more externalizing problems than 6 to 7+ age group. Furthermore, post hoc analysis reveal that younger children have more behavioral problems than the older sample. Along with behavioral problems these children have good social emotional competency and with age the scores on social emotional competence decrease.

**Table 3:** Comparison between Males (n = 206) and Females (n = 220) on internalizing, externalizing and social emotional competence (N = 426).

Scales	Males		Females					*95% CI*		*Cohen’s*
	*M*	*SD*	*M*		*SD*	*t (424)*	*p*	*LL*	*UL*	*d*
IP	58.47	13.00	56.04		12.23	1.98	.047	.02	4.83	0.19
EP	56.82	10.52	58.18		10.90	-1.31	.191	-3.40	.68	0.12
TP	56.90	13.59	58.19		12.59	-1.01	.312	-3.77	1.21	0.09
SEDA	21.93	2.42	22.20		2.47	-1.13	.259	-.73	.198	0.11

IP = internalizing problems, EP = externalizing problems, TP = total behavioral problems. Cohen (1969) [27] classified effect of 0.2 as small, 0.5 as medium, and 0.8 or higher as large.

**Table 4:** Difference among age groups on study variables (N = 426).

	Age group 1 (4–4.11) (n = 26)		Age group 2 (5–5.11) (n = 103)		Age group 3 (6–6.11) (n = 128)		Age group 4 (7–8) (n = 169)					
Variables	M	SD	M	SD	M	SD	M	SD	F	P	ηp^2^	Post hoc Analysis
IP	63.54	9.709	63.30	12.295	56.32	12.830	53.21	11.364	17.856	.000	0.11	1>3, >4; 2>3, >4; 3>4
EP	58.46	7.829	61.01	10.312	58.33	11.228	54.64	10.286	8.404	.000	0.05	2>3, >4
TP	63.42	11.243	63.32	13.253	56.91	13.707	53.65	11.146	14.850	.000	0.09	1>3, >4; 2>3, >4; 3>4
SEDA	23.08	1.262	21.84	2.656	21.59	2.874	22.40	1.989	4.510	.004	0.03	1>2, >3; 2<4; 3<4

IP = internalizing problems, EP = externalizing problems, TP = total behavioral problems. *df* = 3; ηp^2^ = Partial eta squared values are suggestive of significant effect size. Cohen (1969) [27] classified effect of 0.2 as small, 0.5 as medium, and 0.8 or higher as large.

### Social emotional competence and behavioral problems

Tables 5 and 6 present the significant association between SEC and behavioral problems. Child’s higher social emotional competence shows significantly lower level of internalizing and externalizing problems. The multiple linear regression analysis also reveals the impact of behavioral problems on social emotional competence of the children. Observing the findings, it is concluded that behavioural problems together accounts for 16.8% of variance in social emotional competence, with a significant F ratio (*R² =* .028, *F*(2,423) = 6.17, *p* = .002). The behavioural problems are significant negative predictor of SEC in children whereas, externalizing behavioural problems emerge as stronger negative predictor with Beta value (*B =* -.034, *β =* -.150, *p =* .068) indicate that one unit increase in externalizing behavioral problems will result in .34 units decrease in SEC.

**Table 5:** Bivariate correlations between social emotional competence and internalizing, externalizing problems.

Variables	Internalizing Problems	Externalizing Problems
**Social emotional competence**	-.144**	-.168**

** = p < 0.001.

**Table 6:** Multiple linear regression analysis of associations between SEC and Externalizing and internalizing problems.

Variables	B	SE	*Β*	*p-*Value	R^2^	F (2, 423)
**Constant (SEC)**	24.28	.641		.000	.028	6.171**
**Internalizing problems**	-.004	.016	-.022	.785		
**Externalizing problems**	-.034	.019	-.150	.068		

B = “unstandardized regression coefficient”; SE = “Standard error; *β = “*Standardized regression coefficient”; *p-*value = “level of significance;

*** = p < 0.000;

** = p < 0.01;

* = p < 0.05.

## Discussion

The present study was conducted with the primary goal of assessing behavioral problems and its association with social emotional competence in young school children between 4 and 8 years. Findings related to behavioral problems among children revealed alarming borderline and clinical ranges on CBCL scale. The study shows that 41.5% of all children were categorized as “abnormal” as in borderline and clinical ranges, with higher internalizing problems (45.1%) than externalizing problems (39.9%). The prevalence of total problems (41.5%) is similar to the earlier preschool and school children studies conducted in Pakistan such as preschoolers (46.5%, assessed with CBCL) [19] and school children (34.4%, assessed with SDQ) [18] and also to US national survey (41%) [9]. The prevalence was higher than in Norway (7.1%, assessed through SDQ) and Turkey (11.9%, assessed through CBCL) [31]. The differences could be partly explained by the different measurement tools, income levels or varied cultural representations of behavioral and emotional problems in different countries or to the teacher reports, which are considered pervasive in education research and have considerable potential as child assessments [32,33].

Numerous studies have found age and gender differences in the severity and frequency of children’s behavioral problems. However, these observations are rather inconsistent [31,34]. Our study found that females had more externalizing problems such as rule breaking, aggression, thought, and attention problems. While among males, internalizing problems were more common and severe such as anxiety or depression, withdrawn, and somatic problems. It is also worth noting that younger children had overall more behavioral problems than older children. Internalizing problems are more prevalent than externalizing problems in younger children. Furthermore, there is a strong association found between low family income and children’s behavioral problems as consistent in previous studies [18,19].

There are several factors that contribute to these findings with reference to Pakistan. Firstly, child development differs across genders in terms of cognitive, emotional, and social aspects [35,36]. Second, differences in problems may reflect real behavioral differences caused by child-rearing practices that may include social and environmental factors as well as gender role attribution. In Pakistan, trends of gender norms are changing; females are now expected to perform and excel in academia and to represent themselves in society autonomously, instead of limiting to household chores and being dependent on male figures. According to a recent survey in Pakistan, 53% parents support and desire their daughters working [37], indicating that gender related social roles are shifting.

The findings further revealed that the frequency of borderline and clinical behavioral problems on CBCL was quite high in young children. Since the data is collected in Covid-19 pandemic, where schooling, limited social activities, and no outside play rules have already disrupted daily life for school children. These restrictions may have increased children’s behavioral problems. Recent studies have also reflected an increase in emotional and behavioral problems in school aged children around the globe especially during pandemic [21,38].

Another objective of the study was to observe the association between SEC and behavioral problems. Our findings revealed that there is a significant negative correlation between social emotional competence and internalizing and externalizing problems. Children who lack in SEC have more behavioral problems. This suggests immediate need to plan social emotional learning programs at school level to teach emotional understanding, self management, prosocial behaviors, and resilience which may help children to manage their internalizing and externalizing symptoms. Various programs such as PATHS, FRIENDS and Coping Power have established its efficacy in building resilience and reducing behavioral and emotional concerns in children [39–41]. However, it is recommended to include these programs in school curriculums for Pakistani children.

The strength of this study is based on representation of community sample based on cluster sampling that is adequately powered to estimate prevalence of behavioral problems and to make study generalizable. The assessment was done using the formally adapted and locally validated tool of CBCL, which is an internationally established measure.

The research evidence on emotional and behavior problems of young children in Pakistan is scarce. Previous studies’ have focused more on parents’ reports and older children. In Pakistan, the behavior problems of young school children have not been studied for this age group. The present study is unique in this regard because it used teacher reports and sample represented public school’s children from low income group as recommended in a recent telephonic survey [18]. Moreover, results also showed significant abnormal (borderline and clinical) markers in young children, which further required immediate attention and intervention plans at school levels. Studies have [7] established that mental health problems manifest at an early age could endure into adulthood, putting additional strain on the individual, family, friends, and the healthcare system. Therefore, early interventions play a critical role in their development [24].

In our study, there is noticeable correlation found between child’s social emotional competence and behavioral problems. Children who have better social emotional competence levels have showed lower levels of internalizing and externalizing problems. Particularly, children with high levels of externalizing problems predict low social emotional competence than internalizing problems. Externalizing problems are easily observable and noticed by teachers in the classroom environment such as rule breaking, aggressive behavior and hyperactivity and these problems are related with impulsiveness, lack of self control, emotional knowledge and understanding and interpersonal skills.

Additionally, externalizing problems were found strongly related to social emotional competence than internalizing problems in our findings. The possible reason may be related to emotional expression of the child which varies across cultures. In Asian culture, child’s emotional expression varied with familial and environment context [42]. Pakistani children experience difficulty in emotional understanding and regulation as evidenced in recent research [39,40]. Due to lack of adequate emotional management, children may express depressive and somatic complaints as externalizing behaviors such as aggression, disobedience and defiance, which are easily observable and noticeable in child’s behavior in the school environment. Furthermore, younger children showed better social emotional competence than older children. These findings can be better explained with the concept of children’s emotional understanding and development concepts. With growing age, complexity of emotions increased and children are required to attach meanings to emotion, whereas in preschool age, children are developing their ability to regulate and learn emotional expressions without struggling with emotional masking [43–45].Thus, older children may need social emotional skills training to learn complex emotions and emotional regulation with reference to environment, whereas, younger children may need basic lessons of emotional recognition, labeling and knowledge as recommended in previous research [39].

### Limitations and recommendations

The cross-sectional study collected data from public sector schools in Islamabad metropolitan area. Therefore, the generalizability of findings of present study are only for public sector schools. In future work, to increase reliability, data from private schools can be obtained using both parent and self-report measures (where applicable). Later age groups 8–16 years may also be included to perform comparative analysis and predict developmental progression of behavioral problems. Multi-informant data sets with more demographic information shall provide better understanding of cultural factors of behavioral problems. In addition, a nationwide study is suggested to conduct to get more adequate prevalence estimates for Pakistani children. As emotional and behavioral problems in young childhood are likely to persist in adulthood, early identification and assessment would help teachers, clinicians, counselors and parents to take better steps in devising management plans.

## Conclusion

We examined the estimates of behavioral problems in young school children from public schools in Islamabad. As a result, we found alarming frequencies of borderline and clinical ranges of internalizing and externalizing problems and gender differences.

In females, the problem of rule breaking and thought problems in young childhood may be associated with occurrence of externalizing problems such as conduct disorders and disobedience. Such problems in females will be seen as severe stigmatization in our culture.

For males, increased levels of anxiousness, depressed characteristics and emotionally reactivity may be associated with internalizing and emotional problems which is also consistent with the cultural representation of males’ behavior. However, it may lead to more confusion and lack of self awareness.

We conclude that it is necessary for clinicians and teachers to consider the background of behavioral problems at school age and take immediate preventive means to teach coping strategies such as universal social emotional learning programs or targeted interventions. Furthermore, the development of screening system for early intervention is required for school-aged children.
